# Design and Development of Ultrabroadband, High-Gain, and High-Isolation THz MIMO Antenna with a Complementary Split-Ring Resonator Metamaterial

**DOI:** 10.3390/mi14071328

**Published:** 2023-06-29

**Authors:** Ammar Armghan, Khaled Aliqab, Meshari Alsharari, Osamah Alsalman, Juveriya Parmar, Shobhit K. Patel

**Affiliations:** 1 Department of Electrical Engineering, College of Engineering, Jouf University, Sakaka 72388, Saudi Arabia; 2 Department of Electrical Engineering, College of Engineering, King Saud University, P.O. Box 800, Riyadh 11421, Saudi Arabia; 3 Department of Mechanical and Materials Engineering, University of Nebraska-Lincoln, 1400 R St. Nebraska, Lincoln, NE 68588, USA; 4 Department of Computer Engineering, Marwadi University, Rajkot 360003, India

**Keywords:** MIMO, metamaterial, antenna, optimization, THz, ultrabroadband, high gain, high isolation

## Abstract

The need for high-speed communication has created a way to design THz antennas that operate at high frequencies, speeds, and data rates. In this manuscript, a THz MIMO antenna is designed using a metamaterial. The two-port antenna design proposed uses a complementary split-ring resonator patch. The design results are also compared with a simple patch antenna to show the improvement. The design shows a better isolation of 50 dB. A broadband width of 8.3 THz is achieved using this complementary split-ring resonator design. The percentage bandwidth is 90%, showing an ultrabroadband response. The highest gain of 10.34 dB is achieved with this design. Structural parametric optimization is applied to the complementary split-ring resonator MIMO antenna design. The designed antenna is also optimized by applying parametric optimization to different geometrical parameters. The optimized design has a 20 µm ground plane, 14 µm outer ring width, 6 µm inner ring width, and 1.6 µm substrate thickness. The proposed antenna with its broadband width, high gain, and high isolation could be applied in high-speed communication devices.

## 1. Introduction

Antennas are a type of transducer used for communicating wirelessly between two devices. Antennas, which in the past were designed to have a huge aperture size, have now been reduced to small and compact nanoantennas. This reduction in the size of the antenna also extensively reduced its gain; therefore, there is now a need for high-gain compact antennas. There are various ways of improving the gain and bandwidth of an antenna, and one of these ways is to incorporate metamaterials. Today, high-speed communication needs THz antennas to be operated at high speeds. The THz antenna has in recent times been researched by many researchers to be used in high-speed wireless communication devices. The THz antenna offers a higher bandwidth, which could be used to transfer more data at high speeds. The need for high-gain and high-bandwidth THz antennas has increased. The gain and bandwidth of these THz antennas can be improved using metamaterials.

Metamaterials are artificial materials that can be used to improve many parameters of THz antennas [1]. Split-ring resonators or thin wires can be used to create these metamaterials [2]. Complementary split-ring resonators can also be applied to THz antennas to improve their bandwidth and gain [3]. Metamaterial structures can be used to improve the frequency of THz peaks. Simulation results showing a peak enhancement of 0.5 THz were achieved using metamaterial structures [4]. A graphene-material-based metamaterial design has previously been used to improve the gain of the THz antenna design [5]. The design of a microstrip antenna was also presented for THz applications. The antenna was designed with metamaterial loading, offering a metamaterial realization of the circular split-ring resonator. Traditional microstrip patch antennas are produced to be smaller and using more efficient metamaterials [6]. For THz uses, current research delves into the topic of designing adjustable MIMO antennas with superior isolation, both in terms of their laterality and orthogonality placement. It is possible to tune antennas by changing the chemical potential of the patches composed of trapezoidal graphene loaded onto metallic patch antennas. With the help of flawed ground structures, mutual coupling between the MIMO antennas can be reduced, even when they are placed laterally [7]. A triple-band MPA built on a polyimide substrate and integrated with a metamaterial has been presented as a potential solution for future healthcare applications in the terahertz band [8]. An antenna with a tunable resonance frequency via an external voltage was presented through the use of a graphene material. The graphene SRR was used as a metamaterial element in the design. By taking advantage of the fact that the chemical potential of graphene can be altered separately for the patch and the array, it is possible to optimize the material’s properties. A lens could then be incorporated into the design to improve the radiation properties of the proposed layout [9]. MIMO antennas are used in high-speed wireless communication applications [10]. MIMO antennas are applicable in 5G applications [11] and millimeter-wave applications [12].

Today, THz MIMO antennas are used in high-speed communication devices because of their good isolation, high gain, and high bandwidth. MIMO array antennas have been designed with graphene materials, and high isolation has been achieved using a serpentine resonator. The antennas have been applied in THz communication [13]. The two-port MIMO antenna design was created for THz communication where the graphene-based design provides high isolation with simultaneous transmit and receive modes [14]. One other two-port MIMO communication channel design has been used for THz applications, where the light in the design is maintained between MIMO channels. To maximize the system’s throughput and reliability, parallel channels created using appropriately spaced antenna elements have been used, inspired by the principles of diffraction-limited optics [15]. Single-element monopole antennas have been extended into four-port MIMO antennas, and designed with improved isolation for THz applications. The bandwidth was improved using the four-element MIMO antenna design [16]. The graphene-material-based patch antenna design is reconfigurable through the change of the chemical voltage of the graphene material. This reconfigurable MIMO antenna is applicable in THz applications [17]. MIMO THz antennas are also applicable in the design of quantum key distribution. The MIMO antenna design has been shown to have a more powerful distribution compared to the single-element antenna design. The MIMO antenna has one extra secret key component compared to the simple antenna design [18], which is the tapered square patch design used for THz communication. The design is fed through using a microstrip line with a partial ground plane. This design has been shown to be capable of achieving good results for application in high-speed THz communication devices [19]. The MIMO antenna also provides better performance when loaded with metamaterials, which can be realized in the form of different components, such as thin wires, split-ring resonators, complementary split-ring resonators, etc. The improved MIMO antenna is applicable in many GHz [20] and THz applications [21].

The MIMO antenna design has been investigated for use in 5G communication, and has also been presented for integration with portable devices [22]. The use of the broadband THz four-port MIMO antenna covering a broad spectrum of frequencies was presented in [23]. The isolation of the MIMO antenna is important, as all the radiating elements need to be isolated from each other. High isolation is, thus, important, and one such MIMO antenna design with high isolation was presented in [24]. The loading of metamaterial elements onto the MIMO antenna design allows for the tunability of the polarization, which is important when used for differently polarized antennas [7]. The size of the antenna is also important if desired to use in portable applications. One such compact antenna for 5G applications was presented in [25]. High-gain and wideband antennas are applicable in many applications, as they cover most frequency bands for different applications. One such MIMO antenna for achieving a high-gain and wideband response was presented in [26] for vehicular applications.

The optimization of the structural design is essential in achieving the optimized parameters. The different types of optimization that can be applied to these designs are nonlinear parametric optimization and linear parametric optimization [27]. The selection of the optimization algorithm is based on the behavior of the response, whether it be linear or nonlinear [28]. Nonlinear parametric optimization has previously been presented and used to achieve high absorption for the design of a solar absorber [29]. A similar approach could also be applied for the design of an efficient antenna with enhanced parameters, such as the bandwidth, gain, etc. Mutual coupling between patch antenna array elements can be reduced through several approaches. One of the approaches was discussed in detail using a ladder resonator, presented in [30].

The growing demand for high-speed wireless communication devices has led the way toward the design of an antenna that works at the THz frequency. As such, we propose a MIMO antenna design that shows a broadband and high isolation response at the THz frequency range. The proposed antenna is designed with a CSRR-loaded patch antenna. The antenna design comprising a simple microstrip patch is also designed with similar dimensions to show the improvement in the design. Nonlinear parametric optimization is applied to different parameters to achieve the optimal MIMO antenna design. The proposed antenna could be essential for use in high-speed wireless communication devices. We show the design, results, and analysis in the upcoming sections.

## 2. THz MIMO Antenna Design

**Figure 1:**
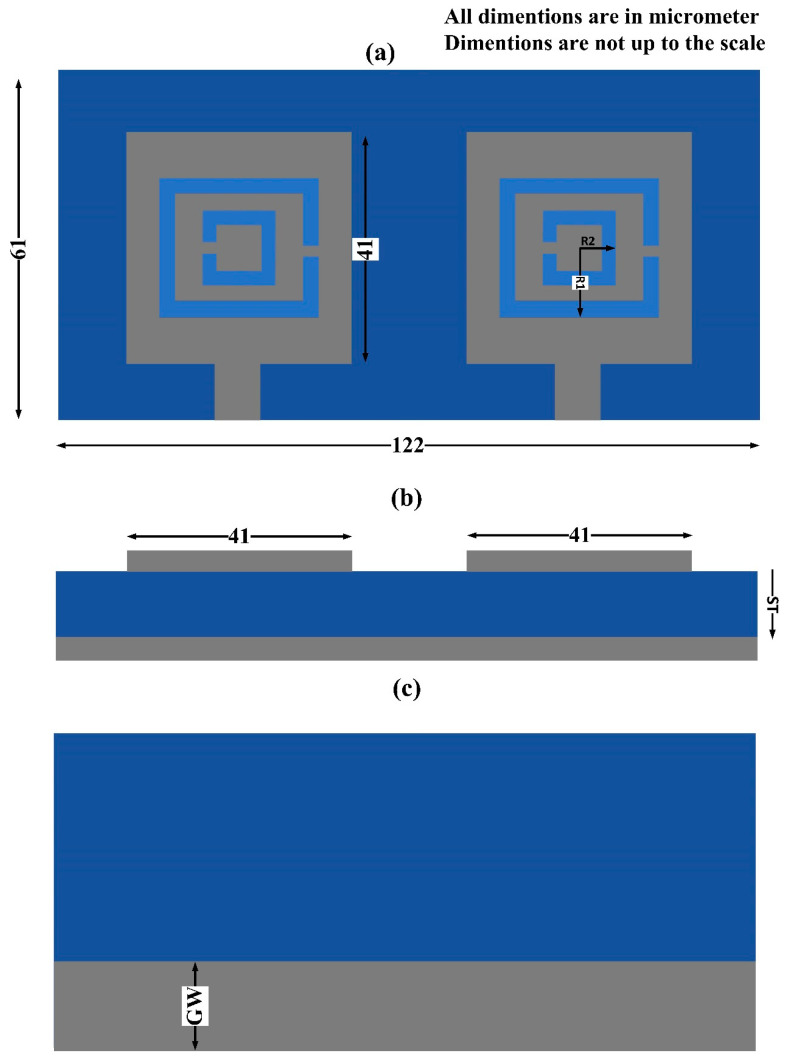
The CSRR metamaterial-based MIMO antenna design. (**a**) Top view of the THz MIMO antenna design showing the complementary split-ring resonator design. The patch was 41 × 41 µm^2^. (**b**) Front view of the THz MIMO antenna design showing the MIM layer design. (**c**) Defected ground plane with 20 µm ground plane width (GW). The substrate and ground plane length was 122 µm. The width of the substrate was 61 µm. The substrate thickness (ST), inner ring width (R_2_), and outer ring width (R_1_) were 1.5 µm, 6 µm, and 14 µm, respectively.

The THz MIMO antenna design was first prepared with a simple square patch, complemented by split-ring resonator etching, and, finally, through preparing the design of the complementary split-ring resonator patch antenna. The design is presented in Figure 1, showing different views for a better understanding of the design. The blue color indicates the dielectric substrate, and the gray part is the metal patch and ground plane. The complementary square split-ring resonator patch was 41 × 41 µm^2^ in size. The two elements of this patch were used to prepare the two-port MIMO antenna. The MIMO antenna was fed through with a matched microstrip line, as shown in Figure 1a. The two-port MIMO antenna was placed over a 122 µm substrate with two resonating elements 20 µm apart from one another. The MIMO antenna was backed with a defected ground structure, which was used to improve the performance of the antenna. The ground was defected through etching a section of the ground plane. The width of the ground plane was then reduced from 61 µm to 20 µm. The thickness of the substrate layer was optimized to 1.5 µm. The optimization results are given in the following results section. The complementary split-ring resonator width was also optimized. The optimized value of the CSRR rings was R1 = 14 µm and R2 = 6 µm.

The antenna parameter calculation mainly depended on the following equation, where the length and width are inversely proportional to the frequency, as shown in Equations (1)–(4):

(1) $$W=\frac{C}{2fr}\sqrt{\frac{2}{\varepsilon r+1}}$$

(2) $$\varepsilon eff=\frac{\varepsilon r+1}{2}+\frac{\varepsilon r-1}{2}{\left[1+12\frac{h}{w}\right]}^{-0.5}$$

(3) $$\frac{\Delta L}{h}=0.412\frac{\left(\varepsilon eff+0.3\right)\left(\frac{w}{h}+0.264\right)}{\left(\varepsilon eff-0.258\right)\left(\frac{w}{h}+0.8\right)}$$

(4) $$L=\frac{1}{2fr\sqrt{\varepsilon eff\mu_{0}\varepsilon_{0}}}-2\Delta$$

The envelope correlation coefficient (ECC) and diversity gain (DG) could be calculated as per Equations (5) and (6) [31].

(5) $$\mathrm{ECC}=\frac{{\left|S_{11}^{*}S_{12}+S_{21}^{*}S_{22}\right|}^{2}}{\left(1-\left({\left|S_{11}\right|}^{2}+{\left|S_{21}\right|}^{2}\right)\right)\left(1-\left({\left|S_{22}\right|}^{2}+{\left|S_{12}\right|}^{2}\right)\right)}$$

The improvement in the SNR of the multiple-element system over a one-element system is referred to as diversity gain (DG). The DG was calculated using the following Equation (6).

(6) $$\mathrm{DG}=10\sqrt{1-|\mathrm{ECC}{|}^{2}}$$

The results of the ECC and DG are discussed further in the results and discussions section.

## 3. THz MIMO Design Results

**Figure 2:**
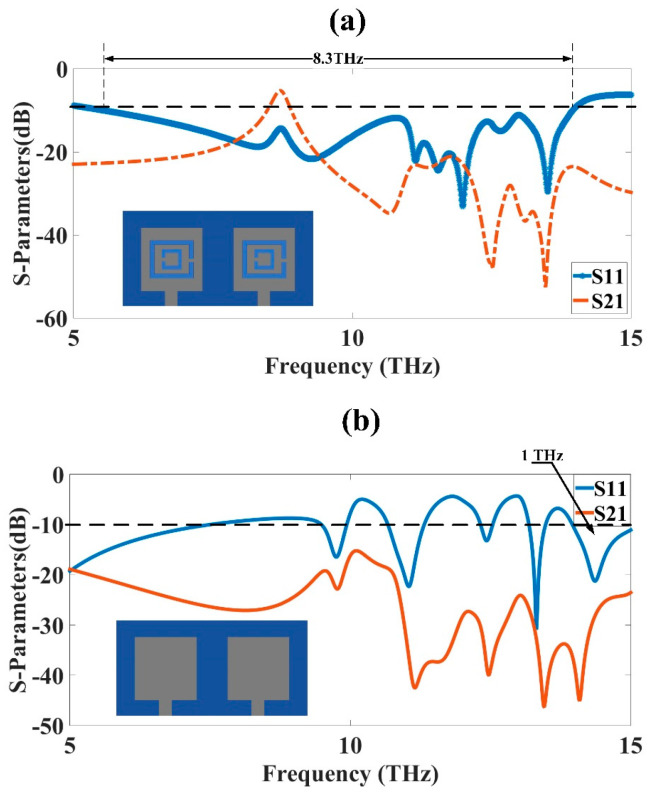
S-parameter results in dB for THz MIMO antenna designs. (**a**) CSRR metamaterial-loaded patch antenna. (**b**) Simple patch design. The patch was 41 × 41 µm^2^ backed with 122 × 61 µm^2^ SiO_2_ substrate. The ground plane and patch were composed of a gold material. The thickness of the ground plane and patch was 500 nm.

**Figure 3:**
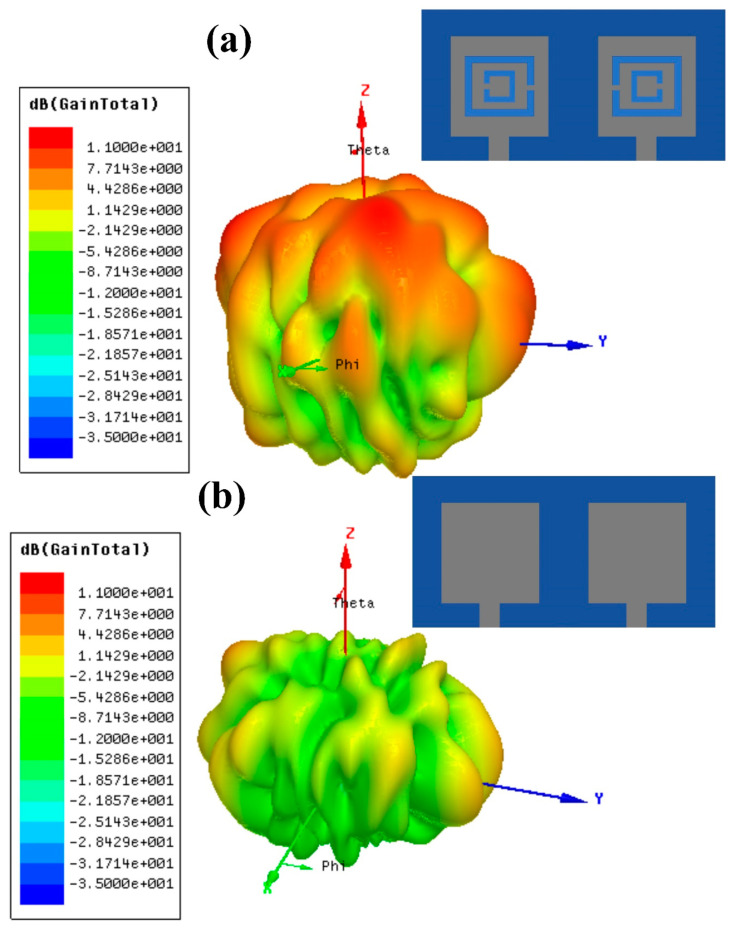
Gain results for both MIMO designs in dB. (**a**) CSRR metamaterial-loaded patch antenna. (**b**) Simple patch design. The highest gain of the CSRR metamaterial-loaded patch design was 10.34 dB, while the simple patch design was 4.18 dB. The metamaterial inclusion improved the gain by more than double its original value. The comparison of the two designs for different antenna parameters is presented in Table 1. The comparison showed that the CSRR metamaterial MIMO antenna design outperformed the simple patch MIMO antenna design.

The design presented in Figure 1 was analyzed to obtain its results for different S-parameters. The square patch design 41 × 41 µm^2^ in size was simulated first, and its response in terms of the S-parameters and gain was obtained. The square patch design was then etched with a split-ring resonator, and a complementary split-ring resonator metamaterial patch design was obtained, given in Figure 1. The new metamaterial design was analyzed and its results were obtained and shown in Figure 2. The results for the simple patch MIMO design were compared with the complementary split-ring resonator metamaterial patch antenna design, with the comparison showing that the metamaterial design had better performance in terms of the S-parameters and gain. The bandwidth obtained was also higher for the metamaterial design. The bandwidth obtained for the CSRR metamaterial patch MIMO antenna design was 8.3 THz, showing its ultrabroadband behavior. This high bandwidth could be used for the development of higher bandwidth high-speed communication system designs. The simple patch MIMO antenna design had the highest bandwidth of 1 THz between 14 THz and 15 THz. The simple MIMO antenna design gave four bands with the highest bandwidths of 1 THz. The highest isolation of approximately 50 dB was achieved for the CSRR metamaterial patch MIMO antenna design. The design’s results were further optimized using changes in various physical parameters, such as substrate height, ground plane width, outer ring width, and inner ring width. The results for the different MIMO parameters, such as ECC and DG, were analyzed and presented in this section. The gain results for the two designs were also analyzed and presented in Figure 3. The antenna designed in this research could be easily fabricated through the use of lithography and by placing the metal patch over the substrate and etching the patch to create a CSRR shape.

The highest gain for the simple patch MIMO antenna design was 4.3 dB, as shown in Figure 3b. The results presented in the figure showed that the gain for the simple patch antenna design was on the lower side. The addition of metamaterial loading improved the gain of the design. The CSRR metamaterial patch MIMO antenna resulted in 10.34 dB, which was greater than the simple patch MIMO design. The increase in the gain was achieved through the etching of the metamaterial in the simple patch design. The etching of the split-ring resonator from the patch changed its permittivity and permeability, which resulted in the improved response of the antenna design.

### 3.1. MIMO Antenna Parameter Analysis

**Figure 4:**
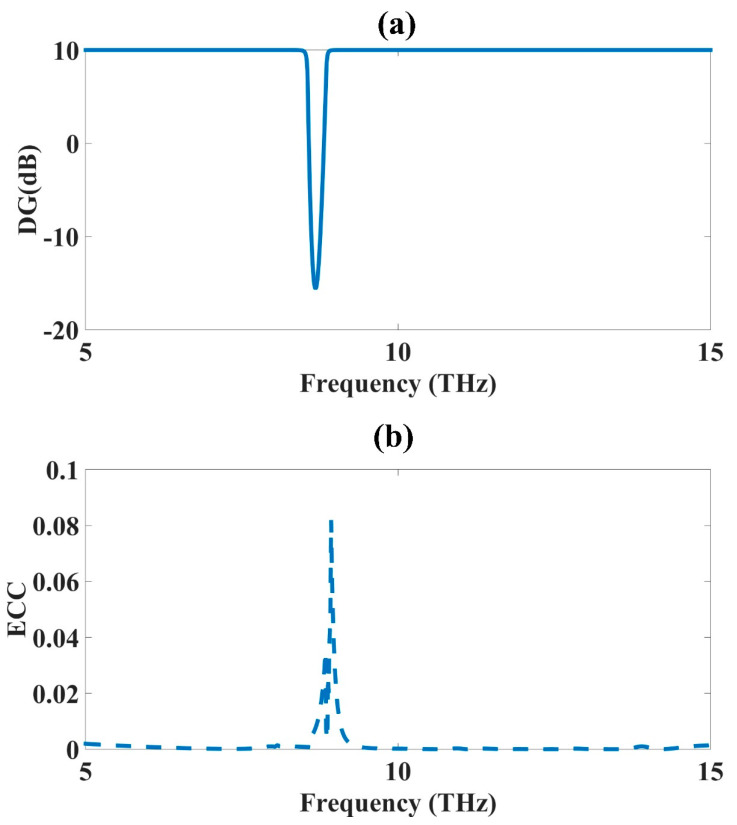
CSRR metamaterial MIMO antenna design parameters: (**a**) DG and (**b**) ECC.

The different MIMO antenna parameters, such as the diversity gain (DG) and envelope correlation coefficient (ECC), were calculated in this section to show their effect on the analyzed spectrum ranging from 5 to 15 THz. Both parameters were analyzed using Equations (5) and (6). The S-parameter results were applied to the equation, and the results achieved for the ECC and DG were given in Figure 4. The ideal value for the DG was 10 dB, but because of losses, it was not achieved fully. The diversity gain reached a value of 10 dB throughout the studied spectrum from 5 to 15 THz, except in some parts of the spectrum at approximately 9 THz. The diversity gain value decayed for some frequencies at approximately 9 THz, which presented the idea that the diversity was inadequate only in this frequency range. The S-parameter curves and isolation between the two antennas was weak around that frequency, which showed that the diversity was also weak, reducing the diversity gain at that point. Similarly, the way the ECC increased at the same frequency also showed that the behavior of the design was adequate in the frequency range of 5 THz to 15 THz. The diversity was good overall for the data transmission and reception in the studied range. The ideal diversity value would have been zero, but there were still some minor values available.

### 3.2. Structural Parameter Optimization

The optimization algorithms could be applied to different structural parameters to obtain optimized parameters that give not only the best results, but also a compact design. The parametric optimization method can be applied to these structural parameters to obtain different optimized parameters. There are two main types of parametric optimization algorithms [32]. The first one is linear parametric optimization, and the second is the nonlinear parametric optimization method [33]. The selection of this method is based on the behavior of the design, whether the behavior of the design is linear or nonlinear. Based on this behavior, the correct type of optimization algorithm can be applied. The results of the reflectance clearly showed that the behavior of the results was nonlinear, and, thus, the nonlinear parametric optimization method could be applied to obtain the optimized structural parameters.

The functions did not behave linearly, which gave this optimization. It had function $f\left(x\right),$ constraint $c_{i}\left(x\right)=1,2,\ldots n,$ or $d_{j}\left(x\right)=1,2,\ldots n,$ which are components of *x* that were nonlinear [34].

The optimization of different structural parameters, such as the substrate height, ground plane width, and CSRR ring widths, was carried out to optimize antenna results, such as the bandwidth and S-parameters.

### 3.3. Substrate Thickness (ST) Optimization

**Figure 5:**
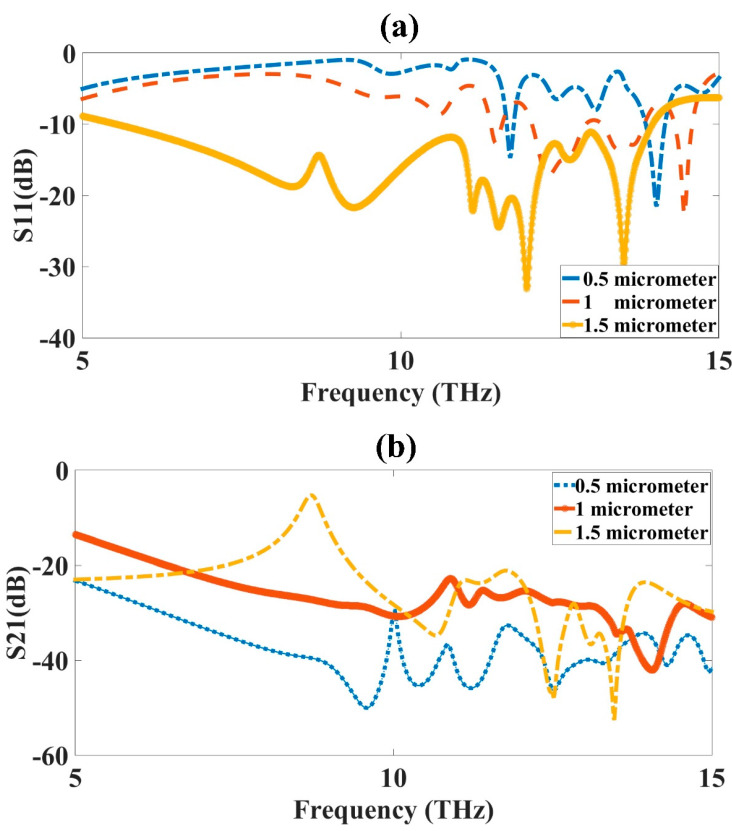
ST variation from 0.5 µm to 1.5 µm. (**a**) S_11_ and (**b**) S_21_. The variation was carried out for a 5 to 15 THz frequency range. The optimized value for the ST was 1.5 µm, shown as the yellow-colored curve in the figure.

The ST optimization was carried out to obtain the highest bandwidth and good results for the MIMO antenna design. The optimization was applied to the CSRR metamaterial-loaded MIMO antenna design. The substrate of the design varied from 0.5 µm to 1.5 µm to observe its effect on the absorption results. The variation was kept at 1.5 µm, because increasing the substrate more than this would increase the overall area of the structure, as well as the cost of the structure, so it was preferential to increase it to a certain limit, keeping it to a 1.5 µm thickness, so that the substrate was kept to a limit, suitable for fabrication and also reducing the cost of the substrate. The variation in the figure clearly showed that the reflectance shown in Figure 5a had the highest bandwidth for the 1.5 µm thickness. The reflectance level was also high for this value only. The yellow color curve showed the reflectance for the 1.5 µm thickness. The red and blue color curves showed a low bandwidth and low reflectance. The transmittance of the design is presented in Figure 5b, also showing that the transmittance results of the yellow curve of the 1.5 µm thickness were −50 dB higher, which showed that there was a 50 dB isolation, which is very good for MIMO antennas. The optimized value of the ST was 1.5 µm.

### 3.4. Ground Layer Width (GW) Optimization

**Figure 6:**
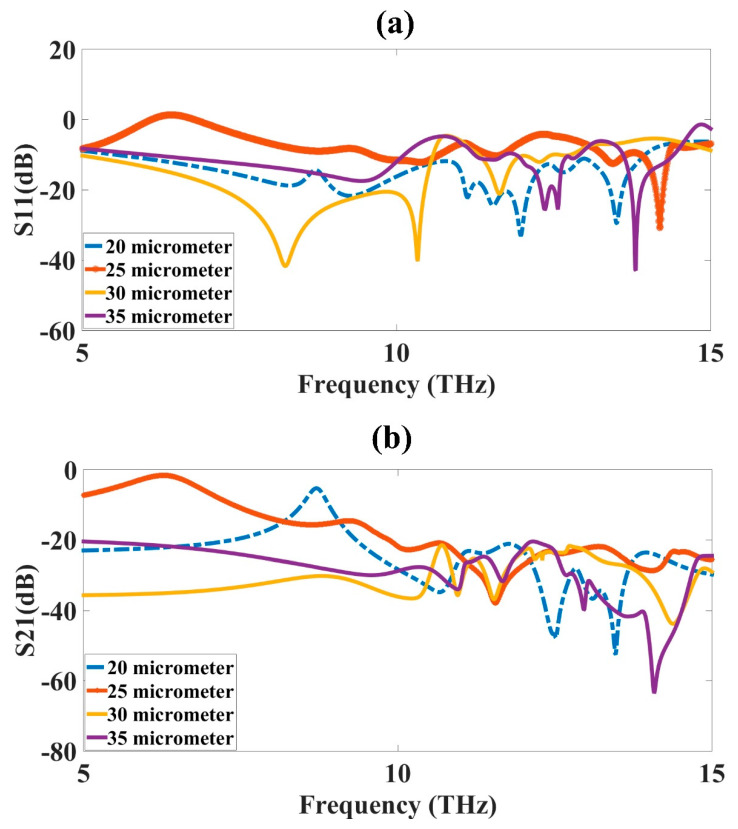
GW variation from 20 µm to 35 µm. (**a**) S_11_ and (**b**) S_21_. The variation was carried out for the 5 to 15 THz frequency range. The optimized value of GW was 20 µm, shown as the blue-colored curve in the figure.

The GW optimization was carried out to obtain the highest bandwidth and good results for the MIMO antenna design. The optimization was applied to the CSRR metamaterial-loaded MIMO antenna design. The ground layer width of the design varied from 20 µm to 35 µm to observe its effect on the absorption results. The variation was kept from 20 µm to 35 µm, because increasing the ground layer width further would have given abrupt results, and the defected ground concept was implemented by etching the part of the ground plane. The variation in the figure clearly showed that the reflectance shown in Figure 6a had the highest bandwidth for the 20 µm ground layer width. The S_21_ results of the design are presented in Figure 6b, showing that for 20 µm, the average value was less than −20 dB, with the highest depth obtained at approximately −50 dB. When the ground layer width was increased to 25, the reflectance showed only one band with a much lower bandwidth, and the transmittance also showed a low isolation compared to the 20 µm results. The increase in the ground layer width did not improve the results any further. Thus, the optimized value of the GW obtained was 20 µm.

### 3.5. Inner Ring Width (R_2_) Optimization

**Figure 7:**
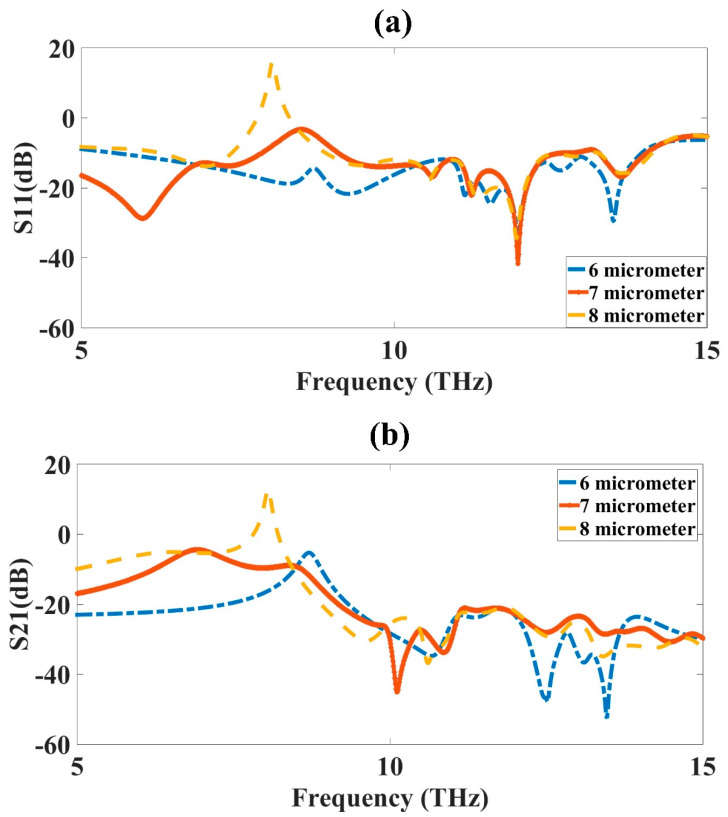
Inner ring width (R_2_) variation from 6 µm to 8 µm. (**a**) S_11_ and (**b**) S_21_. The variation was carried out for 5 to 15 THz frequency range. The optimized value of inner ring width (R_2_) was 6 µm, shown as the blue-colored curve in the figure.

The inner ring width (R_2_) optimization was carried out to obtain the highest bandwidth and good results for the MIMO antenna design. The optimization was applied to the CSRR metamaterial-loaded MIMO antenna design. The inner ring width of the design varied from 6 µm to 8 µm to observe its effect on the absorption results. The variation was kept at 6 µm to 8 µm, because increasing the width of the inner ring further would mix with the outer ring of the CSRR. The change in the ring width would change the capacitance of the metamaterial design. The increase in the width increased the capacitance and degraded the results, as shown in Figure 7. The blue-colored curve in Figure 7a showed the maximum bandwidth of 6 µm. The increase in the inner ring width degraded the response, and there was a reduction in the bandwidth, with more resonating bands available at a lower bandwidth. When the width increased to 8 µm, the result worsened, and there was a mismatch in the power that also resulted in one peak of 20 dB reflectance, which was not valid and, therefore, we could not consider this width for the design of the antenna. The results showed a mismatch as we further increased the inner ring width; thus, only these three values were considered in this result. The S_21_ results also showed that for the increased inner width, the results of the transmittance showed a peak of 20 dB, which was not valid and could not be considered. The optimized value of the inner ring width was 6 µm.

### 3.6. Outer Ring Width (R_1_) Optimization

**Figure 8:**
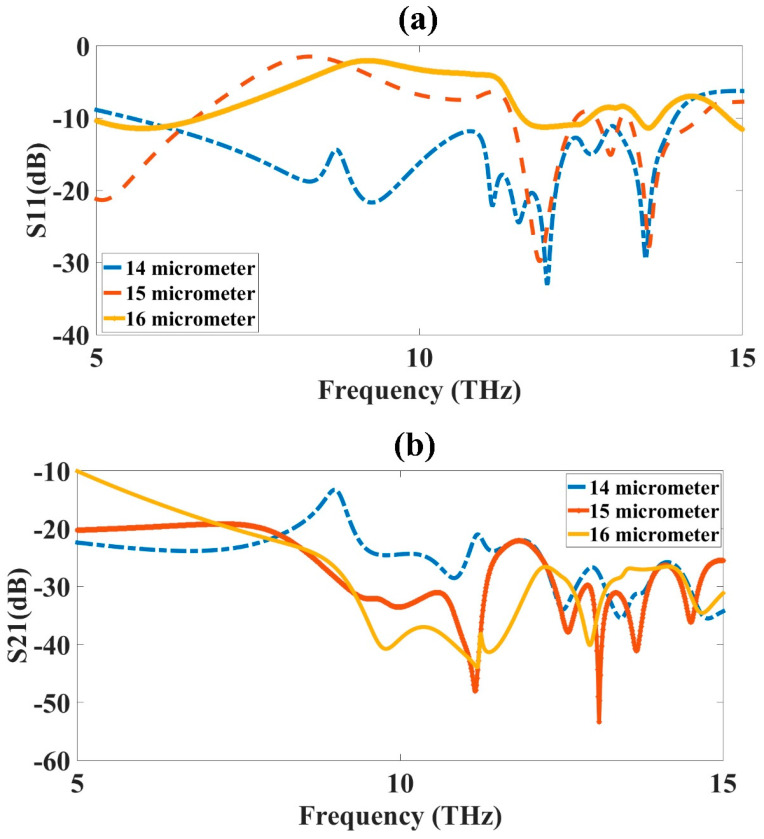
Outer ring width (R_1_) variation from 14 µm to 16 µm. (**a**) S_11_ and (**b**) S_21_. The variation was carried out for 5 to 15 THz frequency range. The optimized value of inner ring width (R_1_) was 14 µm, shown as the blue-colored curve in the figure.

The outer ring width (R_1_) optimization was carried out to obtain the highest bandwidth and good results for the MIMO antenna design. The optimization was applied to the CSRR metamaterial-loaded MIMO antenna design. The outer ring width of the design varied from 14 µm to 16 µm to observe its effect on the absorption results. The variation was kept at 14 µm to 16 µm, because increasing the width of the outer ring would increase the capacitance of the structure. The increase in the width increased the capacitance and degraded the results, as shown in Figure 8. The blue-colored curve in Figure 8a showed the maximum bandwidth of 14 µm. The reflectance for the 14 µm outer width thickness showed better results, giving the maximum bandwidth compared to all the other investigated design lengths. The 15 µm outer ring width was shown with an orange, dashed color plot in the figure, with the plot showing less than −10 dB results for three bands with a maximum bandwidth at approximately 1 THz, which was much lower compared to the 8.3 THz bandwidth of the 14 µm width design. The results were even more degrading for the 16 µm design results, which showed only one band with at approximately −12 dB reflectance, and with a lower bandwidth. Similar results were also obtained for S_21_, with the results presented in Figure 8b. Thus, the optimized outer ring width obtained was 14 µm.

**Figure 9:**
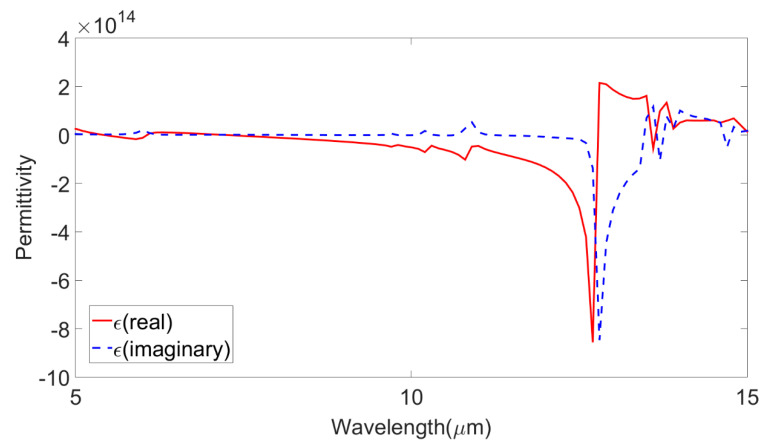
The permittivity curve for the investigated wavelength range of 5 µm to 15 µm. The real part and imaginary parts of permittivity are presented as red-colored and blue-colored dashed lines, respectively.

**Figure 10:**
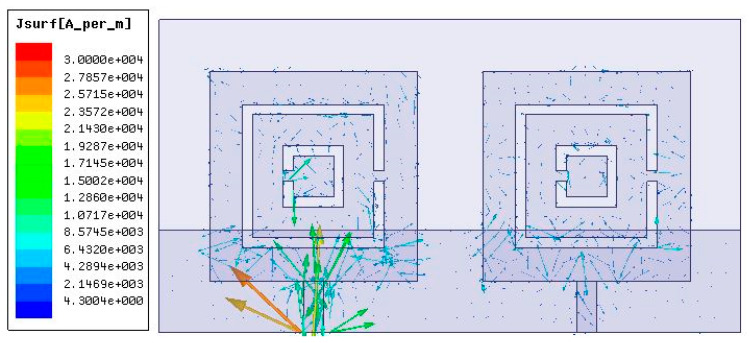
The current distribution in the MIMO antenna design: 3 × 10^4^ A/m.

**Figure 11:**
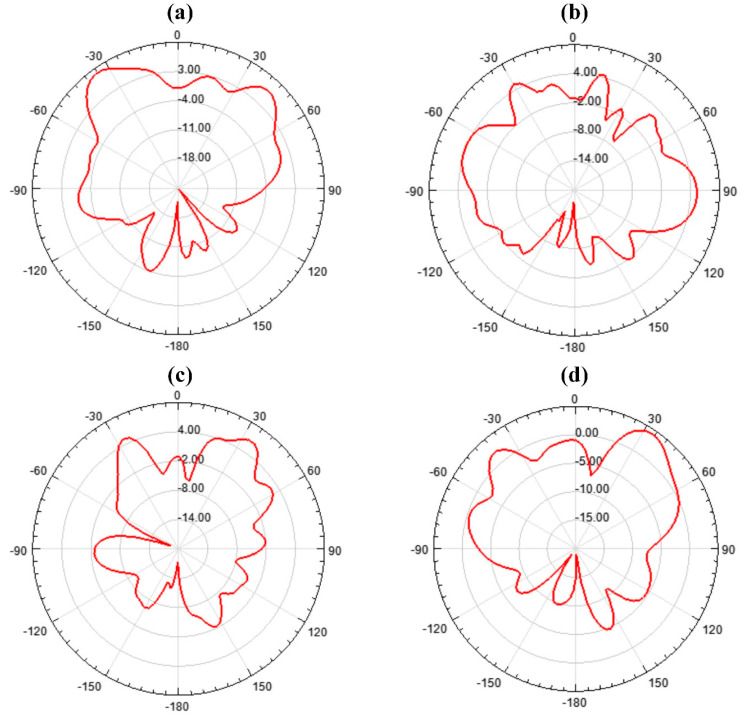
Radiation patterns for different theta values for 10 THz frequency. (**a**) Phi = 45°; (**b**) Phi = 90°; (**c**) Phi = 135°; (**d**) Phi = 180°.

The permittivity, which is very important for metamaterials, was also obtained and presented in Figure 9. Both the real and imaginary parts were presented. The complementary split-ring resonator metamaterial was placed in a two-port network and the permittivity was calculated based on the metamaterial approach given in [35]. To be a metamaterial, its permittivity would have to be negative and in the figure; it was visible that the permittivity attained negative values in the investigated spectrum. The current distribution in the MIMO antenna design is also presented in Figure 10. The current distribution showed the highest current density of 3 × 10^4^ A/m. The 2D radiation pattern plot is also presented in Figure 11 for different phi values. Four different radiation patterns were presented for phi values of 45°, 90°, 135°, and 180°. Radiation patterns were presented for a reference frequency of 10 THz. The radiation patterns for the other frequencies and other phi values could also be similarly achieved.

The THz MIMO antenna design for the CSRR metamaterial patch element and simple patch element was compared with outer designs and presented in Table 2. From the comparison, it was clear that the design had a high bandwidth and high gain. Apart from this, the design of the antenna was simple to fabricate and low cost, as we achieved everything with a two-port MIMO design only compared to a four-port design. Thus, the novelty of the design was shown to be a high bandwidth, high gain, low cost, easy fabrication, and compact size. These were the five novelties associated with our metamaterial MIMO design.

## 4. Conclusions

We compared two THz MIMO antenna designs in our research, namely, a CSRR metamaterial design and a square patch design. The gain and S-parameters were compared for these two designs. The CSRR design showed better performance compared to the other design at a 50 dB isolation, 10.34 dB gain, and 8.3 THz ultrabroadband width. The investigation was carried out for a 5 to 15 THz frequency range. Nonlinear parametric optimization was applied to the ground layer width, substrate thickness, CSRR inner ring width, and CSRR outer ring width. The optimized design parameters were achieved through this optimization. The current distribution and radiation patterns were also presented for the CSRR metamaterial design. The permittivity of the CSRR metamaterial design showed a negative behavior for its real and imaginary parts. The designed MIMO antennas were also compared with other published works. Overall, the proposed MIMO antenna with its high isolation, high gain, and broadband response could be applied in high-speed wireless communication devices.

## Figures and Tables

**Table 1:** CSRR metamaterial MIMO antenna design comparison with simple patch MIMO antenna design.

Design	Bandwidth (THz)	Gain (dB)	Isolation (dB)
CSRR metamaterial MIMO antenna design	8.3	10.34	50
Simple patch MIMO antenna design	1	4.18	45

**Table 2:** CSRR metamaterial MIMO antenna design comparison with simple patch MIMO antenna design and other designs.

Design	Bandwidth (THz)	Gain (dB)	Isolation (dB)
CSRR metamaterial MIMO antenna design	8.3	10.34	50
Simple patch MIMO antenna design	1	4.18	45
[7]	1.4	-	38
[36]	0.6	7.23	55
[37]	0.15	5	50
[15]	-	7.69	-
[38]	1.25	5.72	30
[39]	0.5	3.9	52
[40]	0.12	-	30
[41]	1	-	-

## Data Availability

The data will be made available at a reasonable request to the corresponding author.
